# Intraoperative neurophysiological monitoring in spinal root surgical interventions

**Published:** 2012-11

**Authors:** Alireza Mohammdi, Alireza Jamshidi Fard, Mohsen Dalvandi

**Affiliations:** ^*a*^Department of Neurosurgery, School of Medicine, Arak University of Medical Sciences, Arak, Iran.

## Abstract

**Background:**

Surgical interventions around spinal roots may result in rootlets injury and neurological deficits. Multimodal introperative neurophysiological monitoring (MIOM) can allow for early detection and then reversal of nerve roots potential injuries. These utilities have been currently used to evaluate spinal sensorimotor pathways and nerve root function during posterior spinal approches for transpedicular screw fixations in a rotine base in our setting (Vali-e ASR Hospital, Arak, Iran) since 1998.

**Methods:**

In three consecutive patients (1 male, 16 years old. and 2 female, 17 and 16 years old) kyphoscoliosis corrections, patient’s history, preoperative physical examination and MIOM were performed using a multimodal 40-channel electrophysiologic monitoring system (Nicolet Endeavor, VIASYS Healthcare, 2005, USA). In all cases somatosensory evoked potentials (SSEPs) and F-wave responses corresponding tothe related myotome, were recorded 2 days prior surgery. The SSEPs, bilateral EMGs of paravertebrals and related muscle, stimulus-evoked evoked EMGs by mid-dural spinal stimulation, stimulus-evoked EMGs through root stimulation (pre/post foraminal) were performed when required during different stages of operation procedure. The compound muscle action potentials (CMAPs) were recorded using a pair of 1 Cm2 golden cup surface electrode. A paired-pulse stimulation consisting of two pulses with 2 ms interstimulus interval (ISI), 100-300 µs duration and 10-40 mA intensity was applied through a surgical probe pair electrode touched to the intact dura, over the midline of dorsal column, 2 segments above the involved root, to evoke constant reproducible EMGs in paravertebrals and/or approperiate myotomes of the segment in both lower limbs. Latency of CMAPs were measured for dorsal culumn, foramen entrance and root exit site stimulations. Following the measurements of the F-wave latencies, the central root conduction time and foramenal segments were calculated and used for further monitoring.

The SSEPs of ascending valleis from medial tibial nerve stimulation were recorded at C7 spine and C3´ or C4´of the operation side. A unilateral, sustained loss of 50% of the SSEP amplitude and/or increase by 10% of latency from average values after anesthesia, and SSEP/CMAP waveform change to an asynchronous polyphasic wave, were considered to be pathologic. Drilling and screw positionings were evaluated intraoperatively with standard posteroanterior and lateral radiographs.

**Results:**

None of the 3 patients showed significant change in the SSEPsor post-operative radiculopathies distinct from their preoperative presentations. One hour after the start of surgery, when the spinal column was exposed to the operating theatre temperature, a minimal prolongation of latencies as well as amplitude reduction of SSEPs was observed. The SSEP waveforms were not affected subsequently. A reverse change appeared again after paravertebral muscles stitching at the final hour of surgery.

In our cases, latencies of different root segments were kept intact during the operation and also during the transfer to the recovery room. Propofol or Propofol/Ketamine mixture plus narcotic is suitable to obtain stable reproducible SSEPs and EMGs. Atracurium or other nondepolarizing skeletal muscle relaxants should be avoided. Muscle relaxants application or mean arterial pressure (MAP) below 70 mmHg may cause bilateral reduction or loss of SSEPs and EMGs. There was no postoperative clinically detectable complication.

**Conclusions:**

Findings of our study demonstrated that MIOM should be used in all patients undergoing surgery around spinal roots. Monitoring can practically reduce possibilities of neurological deficit. Futhermore, it can be concluded that the use of SSEPs to evaluate the pedicle screw placement or similar interventions is not an appropriate tool itself, since, , it could be practically limited to sensory fibers of root. In these settings and similar procedures, if IOM is required, alternative multimodal methods with greater sensitivity and efficacy should be explored. To aquire MIOM modalities, close collaboration of an anesthesioloist is nessesary.

**Keywords:**

Spinal surgery, Transpedicular screw, Multimodal intraoperative monitoring


Volume 4, Suppl. 1 Nov 2012
Publisher: Kermanshah University of Medical Sciences
URL: http://www.jivresearch.org
Copyright: (cc) BY

**Paper No. 84**

### Intraoperative neurophysiological monitoring in spinal root surgical interventions

Alireza Mohammdi [a], Alireza Jamshidi Fard [a,*], Mohsen Dalvandi [a]

[a] Department of Neurosurgery, School of Medicine, Arak University of Medical Sciences, Arak, Iran.

### Abstract:

**Background:** Surgical interventions around spinal roots may result in rootlets injury and neurological deficits. Multimodal introperative neurophysiological monitoring (MIOM) can allow for early detection and then reversal of nerve roots potential injuries. These utilities have been currently used to evaluate spinal sensorimotor pathways and nerve root function during posterior spinal approches for transpedicular screw fixations in a rotine base in our setting (Vali-e ASR Hospital, Arak, Iran) since 1998.

**Methods:** In three consecutive patients (1 male, 16 years old. and 2 female, 17 and 16 years old) kyphoscoliosis corrections, patient's history, preoperative physical examination and MIOM were performed using a multimodal 40-channel electrophysiologic monitoring system (Nicolet Endeavor, VIASYS Healthcare, 2005, USA).

In all cases somatosensory evoked potentials (SSEPs) and F-wave responses corresponding tothe related myotome, were recorded 2 days prior surgery. The SSEPs, bilateral EMGs of paravertebrals and related muscle, stimulus-evoked evoked EMGs by mid-dural spinal stimulation, stimulus-evoked EMGs through root stimulation (pre/post foraminal) were performed when required during different stages of operation procedure. The compound muscle action potentials (CMAPs) were recorded using a pair of 1 Cm2 golden cup surface electrode. A paired-pulse stimulation consisting of two pulses with 2 ms interstimulus interval (ISI), 100-300 µs duration and 10-40 mA intensity was applied through a surgical probe pair electrode touched to the intact dura, over the midline of dorsal column, 2 segments above the involved root, to evoke constant reproducible EMGs in paravertebrals and/or approperiate myotomes of the segment in both lower limbs. Latency of CMAPs were measured for dorsal culumn, foramen entrance and root exit site stimulations. Following the measurements of the F-wave latencies, the central root conduction time and foramenal segments were calculated and used for further monitoring.

The SSEPs of ascending valleis from medial tibial nerve stimulation were recorded at C7 spine and C3´ or C4´of the operation side. A unilateral, sustained loss of 50% of the SSEP amplitude and/or increase by 10% of latency from average values after anesthesia, and SSEP/CMAP waveform change to an asynchronous polyphasic wave, were considered to be pathologic. Drilling and screw positionings were evaluated intraoperatively with standard posteroanterior and lateral radiographs.

**Results:** None of the 3 patients showed significant change in the SSEPsor post-operative radiculopathies distinct from their preoperative presentations. One hour after the start of surgery, when the spinal column was exposed to the operating theatre temperature, a minimal prolongation of latencies as well as amplitude reduction of SSEPs was observed. The SSEP waveforms were not affected subsequently. A reverse change appeared again after paravertebral muscles stitching at the final hour of surgery.

In our cases, latencies of different root segments were kept intact during the operation and also during the transfer to the recovery room. Propofol or Propofol/Ketamine mixture plus narcotic is suitable to obtain stable reproducible SSEPs and EMGs. Atracurium or other nondepolarizing skeletal muscle relaxants should be avoided. Muscle relaxants application or mean arterial pressure (MAP) below 70 mmHg may cause bilateral reduction or loss of SSEPs and EMGs. There was no postoperative clinically detectable complication.

Injury&Violence
Volume 4, Suppl. 1 Nov 2012
Publisher: Kermanshah University of Medical Sciences
URL: http://www.jivresearch.org
Copyright: (cc) BY

**Conclusions:** Findings of our study demonstrated that MIOM should be used in all patients undergoing surgery around spinal roots. Monitoring can practically reduce possibilities of neurological deficit. Futhermore, it can be concluded that the use of SSEPs to evaluate the pedicle screw placement or similar interventions is not an appropriate tool itself, since, , it could be practically limited to sensory fibers of root. In these settings and similar procedures, if IOM is required, alternative multimodal methods with greater sensitivity and efficacy should be explored. To aquire MIOM modalities, close collaboration of an anesthesioloist is nessesary.

### Key words:

***Spinal surgery, Transpedicular screw, Multimodal intraoperative monitoring***

*** Corresponding Author at:**
***Alireza Jamshidi Fard:*** *Department of Neurosurgery, School of Medicine, Arak University of Medical Science, Arak, Iran, Tell: +98 9181616251, Email: arjfard@yahoo.com,* ***(Jamshidi Fard A.).***

